# Resistome in the indoor dust samples from workplaces and households: a pilot study

**DOI:** 10.3389/fcimb.2024.1484100

**Published:** 2024-12-03

**Authors:** Eva Klvanova, Petra Videnska, Vojtech Barton, Jan Bohm, Petra Splichalova, Viktorie Koksova, Milan Urik, Barbara Lanickova, Roman Prokes, Eva Budinska, Jana Klanova, Petra Borilova Linhartova

**Affiliations:** ^1^ RECETOX, Faculty of Science, Masaryk University, Brno, Czechia; ^2^ Department of Pediatric Otorhinolaryngology, University Hospital Brno, Brno, Czechia; ^3^ Department of Pediatric Otorhinolaryngology, Faculty of Medicine, Masaryk University, Brno, Czechia; ^4^ Department of Neonatology, University Hospital Brno, Brno, Czechia; ^5^ Department of Gynecology and Obstetrics, University Hospital Brno, Brno, Czechia; ^6^ Department of Gynecology and Obstetrics, Faculty of Medicine, Masaryk University, Brno, Czechia; ^7^ Department of Atmospheric Matter Fluxes and Long-range Transport, Global Change Research Institute of the Czech Academy of Sciences, Brno, Czechia

**Keywords:** antibiotic resistance gene, indoor environment, microbiome, antimicrobial resistance, hospital

## Abstract

The antibiotic resistance genes (ARGs) limit the susceptibility of bacteria to antimicrobials, representing a problem of high importance. Current research on the presence of ARGs in microorganisms focuses mainly on humans, livestock, hospitals, or wastewater. However, the spectrum of ARGs in the dust resistome in workplaces and households has gone relatively unexplored. This pilot study aimed to analyze resistome in indoor dust samples from participants’ workplaces (a pediatric hospital, a maternity hospital, and a research center) and households and compare two different approaches to the ARGs analysis; high-throughput quantitative PCR (HT-qPCR) and whole metagenome shotgun sequencing (WMGS). In total, 143 ARGs were detected using HT-qPCR, with ARGs associated with the macrolides, lincosamides, and streptogramin B (MLSB) phenotype being the most abundant, followed by MDR (multi-drug resistance) genes, and genes conferring resistance to aminoglycosides. A higher overall relative quantity of ARGs was observed in indoor dust samples from workplaces than from households, with the pediatric hospital being associated with the highest relative quantity of ARGs. WMGS analysis revealed 36 ARGs, of which five were detected by both HT-qPCR and WMGS techniques. Accordingly, the efficacy of the WMGS approach to detect ARGs was lower than that of HT-qPCR. In summary, our pilot data revealed that indoor dust in buildings where people spend most of their time (workplaces, households) can be a significant source of antimicrobial-resistant microorganisms, which may potentially pose a health risk to both humans and animals.

## 1 Introduction

Antimicrobial resistance (AMR), i.e., the ability of microorganisms to persist or grow in the presence of drugs designed to inhibit or kill them, poses one of the greatest current threats to global health (Kim and Cha, 2021). AMR occurs in microorganisms naturally, but overuse of antimicrobial agents (antibiotics, antifungals, antivirotics, and antiparasitics) in both humans and animals leads to a significant increase in the number of resistant strains and, in effect, to longer hospital stays, higher medical costs, and increased mortality (Murray et al., 2022).

Acquired antibiotic resistance (AR) arises either as a result of gene mutations in bacterial DNA, or by acquiring an antibiotic resistance gene (ARG) from another bacterium. Mobile genetic elements (MGEs), such as plasmids, transposons, or integrons, contribute to the spread of ARGs across different bacterial genera (Acman et al., 2022). Beside bacterial phyla, some fungal phyla were also considered as ARGs-carrying pathogens (Wang et al., 2023). ARGs can be classified into several categories based on the class of antibiotics they confer resistance to, i.e., aminoglycosides (e.g., *aac*, *ant*, *aph*), beta-lactams (e.g., *blaTEM*, *blaOXA*, *blaAmpC*), multidrug (e.g., *mexA*, *oprJ*, *acrAB-tolC*), macrolides (e.g., *erm*, *mef*, *mph*), tetracyclines (e.g. *tetA*, *tetM*, *tetX*), phenicol (e.g., *cat*, *floR*, *cmlA*), quinolones (e.g., *qnr*, *gyrA*, *norA*), sulfonamides (e.g., *sul1*, *sul2*, *folP*), trimethoprim (e.g., *dfrA1*, *drfA5*, *dfrB*), vancomycin (e.g., *vanA*, *vanB*, *vanC*), and others, including, e.g. colistin (e.g., *mrc1*, *mrc2*). In addition, ARGs encode resistance to macrolides, lincosamides, and streptogramin B (so-called MLSB phenotype), characterized by high-level cross-resistance to these antibiotics (e.g., *ermA*, *ermB*, *ermC*) (Martínez-Martínez et al., 1998; Potrykus and Wegrzyn, 2001; Frye and Jackson, 2013; Wüthrich et al., 2019; Abdelrahman et al., 2020; Schwendener et al., 2020; Zhang et al., 2023; Puangseree et al., 2024; Smith et al., 2024).

The resistome, a collection of all ARGs and MGEs within a specific sample, has mostly been investigated in food-producing animals and human patients, especially in fecal samples (Forslund et al., 2013; Pal et al., 2016; Mencía-Ares et al., 2020; Wang et al., 2020; Nielsen et al., 2021). The main factors contributing to the structure of fecal (and, inherently, human gut) resistome include the administration of antimicrobials, health status, and diet (Forslund et al., 2014; Palleja et al., 2018; Willmann et al., 2019; Oldenburg et al., 2020; Tavella et al., 2021; Stege et al., 2022). The resistome of human cavities such as the ears, nose, and oral cavity has been less studied compared to the gut resistome (Allemann et al., 2019; Carr et al., 2020; Hullegie et al., 2021; Sukumar et al., 2023). However, given that these cavities serve as primary gateways for pathogens and are prone to infections, understanding their resistomes is crucial for human health. Notably, these cavities are in direct contact with the surrounding environment (water, air, and dust particles), which can influence their microbial communities, including their resistomes (Bich et al., 2019; Sun et al., 2020a; Wang et al., 2021; Gwenzi and Siyawamwaya, 2022). Hospitals and agricultural farms represent high-risk environments due to the extensive use of antimicrobials. These environments contribute to the release of antimicrobials and associated AR bacteria into the surrounding ecosystems, e.g. through wastewater. While the presence of resistance determinants in environmental bacteria does not inherently threaten human health, their transfer to human-associated pathogens, via MGEs, could result in significant public health challenges underscoring the importance of studying the human exposure to environmental resistomes (Peterson and Kaur, 2018; Kunhikannan et al., 2021).

Indoor dust, a mixture of particulate matter and biological and chemical pollutants, to which humans living in urban areas are exposed daily, represents a great reservoir of microorganisms and ARGs (Escudeiro et al., 2019; Feng et al., 2022). Since hospitals are an important breeding ground for the development and spread of AR bacteria, the resistome in hospital dust and possible routes of transmission of ARGs across hospital environments were investigated in many previous studies (Chng et al., 2020; Li et al., 2021; Bai et al., 2022; Wu et al., 2022). However, little is known of other important indoor environments where people spend the majority of their lifetime, such as workplaces and households (Gupta et al., 2019; Shan et al., 2019; Ding et al., 2020; Wu et al., 2022). Indoor environments with higher densities of people, such as malls, office buildings, and educational facilities, may be significant reservoirs for ARGs. The high density of people can facilitate greater microbial exchange and enhance the persistence and spread of resistant strains. Thus, while higher microbial diversity can sometimes act as a barrier to the spread of resistance, in densely populated indoor settings, ARGs can accumulate and disseminate through increased human contact and microbial interaction (Hewitt et al., 2012; Zhao et al., 2021; Li et al., 2022; An et al., 2023; Klümper et al., 2024). Moreover, some antimicrobials present in cleaning products used in indoor environments, such as triclosan, are associated with a higher prevalence of specific ARGs in indoor dust (Hartmann et al., 2016). Nevertheless, data comparing the resistomes from workplaces and households, including high-risk hospital settings, is still limited.

In our pilot study, we aimed to analyze the resistomes in paired indoor dust samples collected from three workplaces (a pediatric hospital, a maternity hospital, and offices in a research center) and households of employees of these institutions using (i) high-throughput quantitative PCR (HT-qPCR) techniques and (ii) whole metagenome shotgun sequencing (WMGS). We intended to compare the results obtained by these techniques to investigate their suitability (based on the method sensitivity) for the analysis of ARGs in indoor dust.

## 2 Materials and methods

This brief research report is based on dust samples presented in our previous article containing a detailed description of sampling, DNA extraction, and 16S rRNA sequencing library preparation and analysis (Konecna et al., 2023). In brief, samples were collected using a vacuum cleaner and sampling head with filter paper. DNA from homogenized samples was isolated using DNeasy PowerLyzer PowerSoil kit (QIAGEN, GER). The DNA quality was checked using gel electrophoresis and concentration was measured fluorimetrically using the Quant-iT dsDNA High-Sensitivity Assay Kit (Thermo Fisher Scientific, USA).

Due to the required minimal DNA concentration (10 ng/μl) and volume (100 μl) for ARGs analysis by public service based on the HT-qPCR technique (Resistomap Oy, Helsinki, Finland), it was necessary to pool samples. Hence, we pooled extracted DNA from indoor dust samples separately for each of the three workplaces (ENT, pediatric hospital; NEO, maternity hospital; and RCX, research center) and for households of workers from these workplaces (in total, 22 + 22 samples pooled into 3 + 3 pooled samples), see the workflow in **Supplementary Figure S1**. Specifically, three pooled represented indoor dust samples from the three workplaces (ENT Work, NEO Work, and RCX Work), while the other three pooled samples originated from the homes of the employees working at these workplaces (NEO Home, ENT Home, and RCX Home). HTq-PCR and WMGS analyses were performed on these six paired pools. Each pooled sample consisted of at least six individual samples number of samples in each pool is shown in **Supplementary Table S1**.

### 2.1 HT-qPCR – Resistomap

For our purposes, we selected 247 ARGs (**Supplementary Table S2** with used primers) for HT-qPCR analysis using a SmartChip Real-time PCR system (TaKaRa Bio, Japan) to be examined by Resistomap Oy (Stedtfeld et al., 2018). Currently, Resistomap has been widely used by many authors for ARGs analysis in multiple environments, including aquatic systems, agriculture, hospitals, or human gut helping track the spread of ARGs globally (Bagra et al., 2024; Daw Elbait et al., 2024; Klümper et al., 2024; Samarra et al., 2024). The service Resistomap provided us with raw data containing cycle threshold (Ct) and melting temperature (Tm) values for each gene. All subsequent data analysis and calculations were performed in-house. Only genes with Ct ≤ 28 in at least 2 of 3 replicates were used in analysis. The relative quantification was calculated using the formula: 2^-ΔCt^. The ΔCt was based on the abundance of the genes relative to the amount of gene for 16S rRNA in each sample (ΔCt = Ct of analyzed genes – Ct of gene for 16S rRNA).

### 2.2 WMGS and ARGs identification

Sequencing libraries were prepared using the QIAseq FX DNA lib kit (QIAGEN, USA) following the manufacturer’s instructions. The input of DNA was 10 ng; for all samples, the initial time of fragmentation with an enhancer was 5 min. The quality of all libraries was determined using the TapeStation and its D5000 ScreenTape kit (Agilent Technologies, USA). The concentration was measured using the KAPA Library Quantification Complete Kit (Roche, USA) and the LightCycler 480 Instrument (Roche, USA). Based on these concentrations, the final library was pooled. The quality of the final library was checked using the same methods as employed for individual libraries. The indoor dust samples were sequenced using the NextSeq 550 system and 500/550 High Output Kit v2.5 (300 cycles) according to the manufacturer’s instructions (Illumina, USA).

The raw sequencing data were subjected to quality assessment using FastQC (version 0.11.9) (Andrews, 2010) and the reports were consolidated with MultiQC (version 1.19) (Ewels et al., 2016). Quality control was performed using fastp (version 0.23.3) (Chen et al., 2018) with a mean quality cut-off of 25 and a minimum read length of 50 bp, which was sufficient for ARGs detection. Adapters were automatically detected and removed by the same tool.

For the description of the microbiome in pooled samples, taxonomic profiling of the preprocessed data was carried out using Kraken2 (version 2.1.3) (Wood et al., 2019, p. 2) with the quick flag enabled, utilizing the complete nt database (version nt_20230502).

The contig assembly was executed using the nf-core/mag (version 2.5.1) (Krakau et al., 2022) publicly available and curated pipeline with MEGAHIT (version 1.2.9) as the assembler. The assembled contigs were subsequently analyzed for ARGs using the nf-core/funcscan (version 1.1.4) publicly available and curated pipeline (Yates et al., 2023), incorporating the ABRicate (version 1.0.1) and AMRFinderPlus (version 3.10.42, database version 2022-10-11.2) tools. The results were consolidated using hAMRonization (version 1.1.1). Bioinformatics analysis parameters are shown in **Supplementary Figure S2**.

### 2.3 Statistical analysis

All statistical analyses were performed in R version 4.1.2 (2021-11-01) (R Core Team, 2021). The analyses were solely descriptive, without any inferential tests.

For the HT-qPCR – Resistomap analysis, a radar chart of relative quantifications of ARGs grouped by categories based on the main classes of antibiotics and other genes (e. g., encoding multidrug efflux pumps, resistance to antiseptics, or mercury) to which they confer resistance was created using the fmsb package v0.7.6 (Nakazawa, 2024). A heatmap of the relative quantifications of ARGs from HT-qPCR – Resistomap was created using the ComplexHeatmap package v2.10.0 (Gu et al., 2016). The data were scaled by the minimal non-zero value and transformed using total sum scaling (TSS). For heatmap only 25 genes with highest total relative quantification across all samples and/or those found in both the HT-qPCR – Resistomap and WMGS data were included in the final heatmap. In the WMGS analysis, reads were extracted at both the domain and genera levels and bar plots were generated using the ggplot2 package v3.5.1 (Wickham, 2016).

## 3 Results

Most participants (73%) lived in flats with a floor area of more than 70 m^2^. On average, there were three occupants per household. Three participants, one from each workplace, owned a dog. Average air humidity during sampling was higher in households (58.1%) than in workplaces (45.6%), while the average temperature was lower in households (22.3°C) than in workplaces (26.5°C). Air conditioning was present in 4 out of 22 households. More characteristics of the sampling locations are provided in **Supplementary Table S1** and were previously published by our team (Konecna et al., 2023).

**Figure 1:**
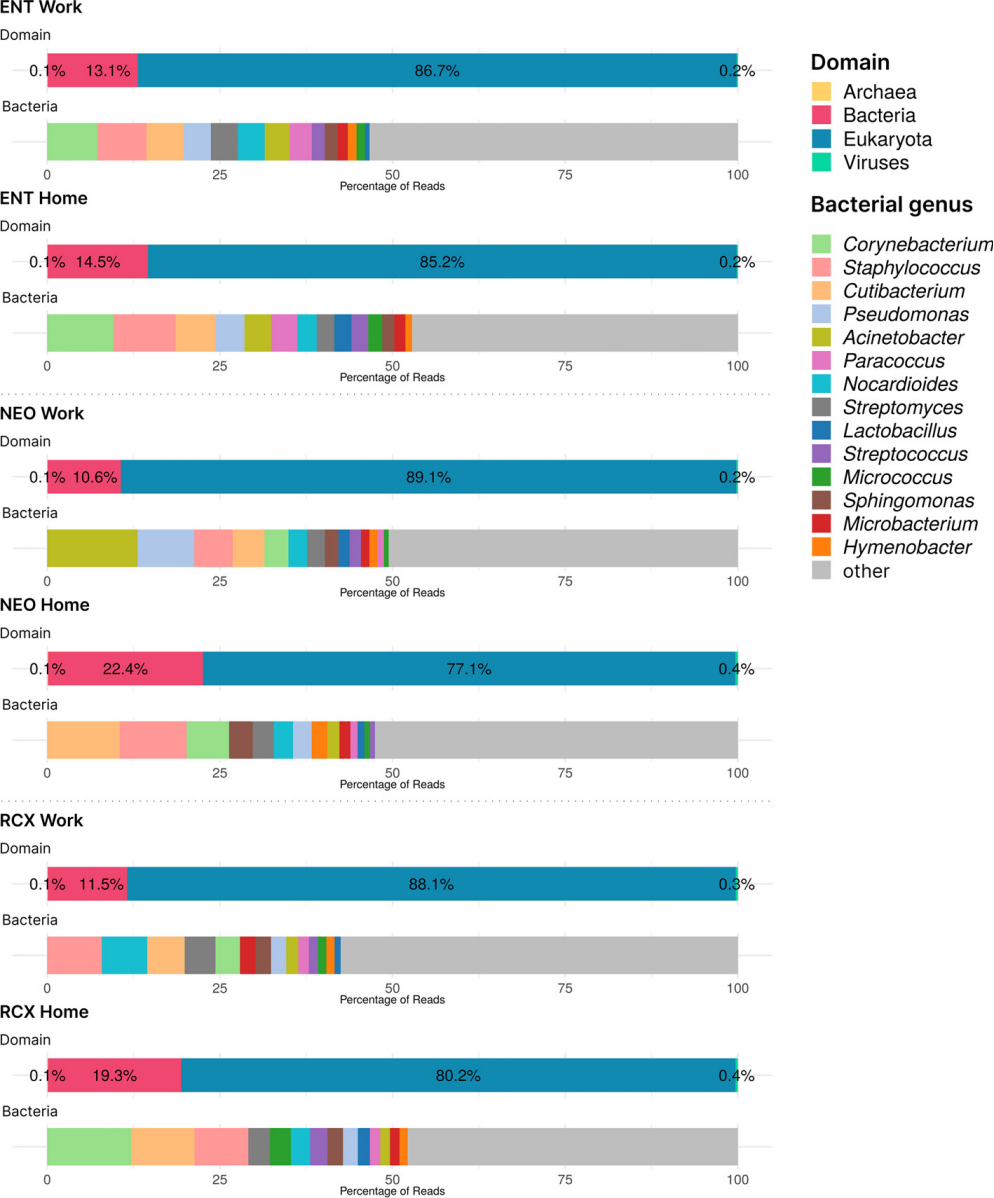
Barplots of DNA taxonomical compositions of the pooled samples from the three workplaces (Work) and respective households (Home) at the domain level and genus level for Bacteria obtained by WMGS. Note the low levels of Archaea and Viruses DNA (< 1%). ENT, pediatric hospital; NEO, maternity hospital; and RCX, research center; WMGS, whole metagenome shotgun sequencing.

**Supplementary Table S1** also presents the numbers of reads from the WMGS analysis (range 2,206,042**–**10,517,515 reads per pooled sample). The WMGS analysis revealed that the six pooled samples predominantly contained eukaryotic (predominantly human) DNA, with a mean relative abundance of 84.1%. A higher relative abundance of human DNA was observed in workplace indoor dust samples compared to household samples. In addition to human DNA, dust samples from households contained higher amounts of DNA from dogs, pines, and spruces compared to samples from workplaces. In the kingdom *Fungi*, the genera *Alternaria*, *Cladosporium*, and *Aspergillus* were the most abundant across all pooled samples. Additionally, other fungi considered to be potential human pathogens, such as the genera *Malassezia*, *Trichoderma*, *Penicillium*, and *Candida*, were also represented. The relative abundance of bacterial DNA in the pooled dust samples ranged from 10.6% to 22.5%. *Corynebacterium*, *Cutibacterium*, and *Staphylococcus* were the most abundant genera in household dust samples, with relative abundances within the Bacteria domain of ≥ 5% (comprising 25.9% of bacterial reads in these samples). Workplace samples, however, exhibited greater diversity, particularly in the environment of NEO, which showed high relative abundances of the genera *Acinetobacter* and *Pseudomonas*. In addition, DNA from the Archea and Viruses domains was found in these indoor dust samples (albeit in very low relative abundances, approximately 0.1% and 0.3% of all reads, respectively). Of Archaea, all genera with relative abundances higher than 3% across all samples belonged to the Halobacteria class (namely *Halorubrum*, *Halobaculum*, *Natrinema*, *Halobacterium*, *Halorussus*, *Halovivax*, and *Haloterrigena).* The Halobacteria class was responsible for 27.6% of archaeal reads. The most abundant viruses with relative abundances of at least 5% across all samples were members of the genera *Lentivirus*, *Betacoronavirus*, *Salasvirus, Coguvirus*, and *Ranavirus*, covering together 42% of the reads in the domain Viruses. The domain composition and relative abundances of the top 15 bacterial genera across all pooled samples are presented in **Figure 1**.

**Figure 2:**
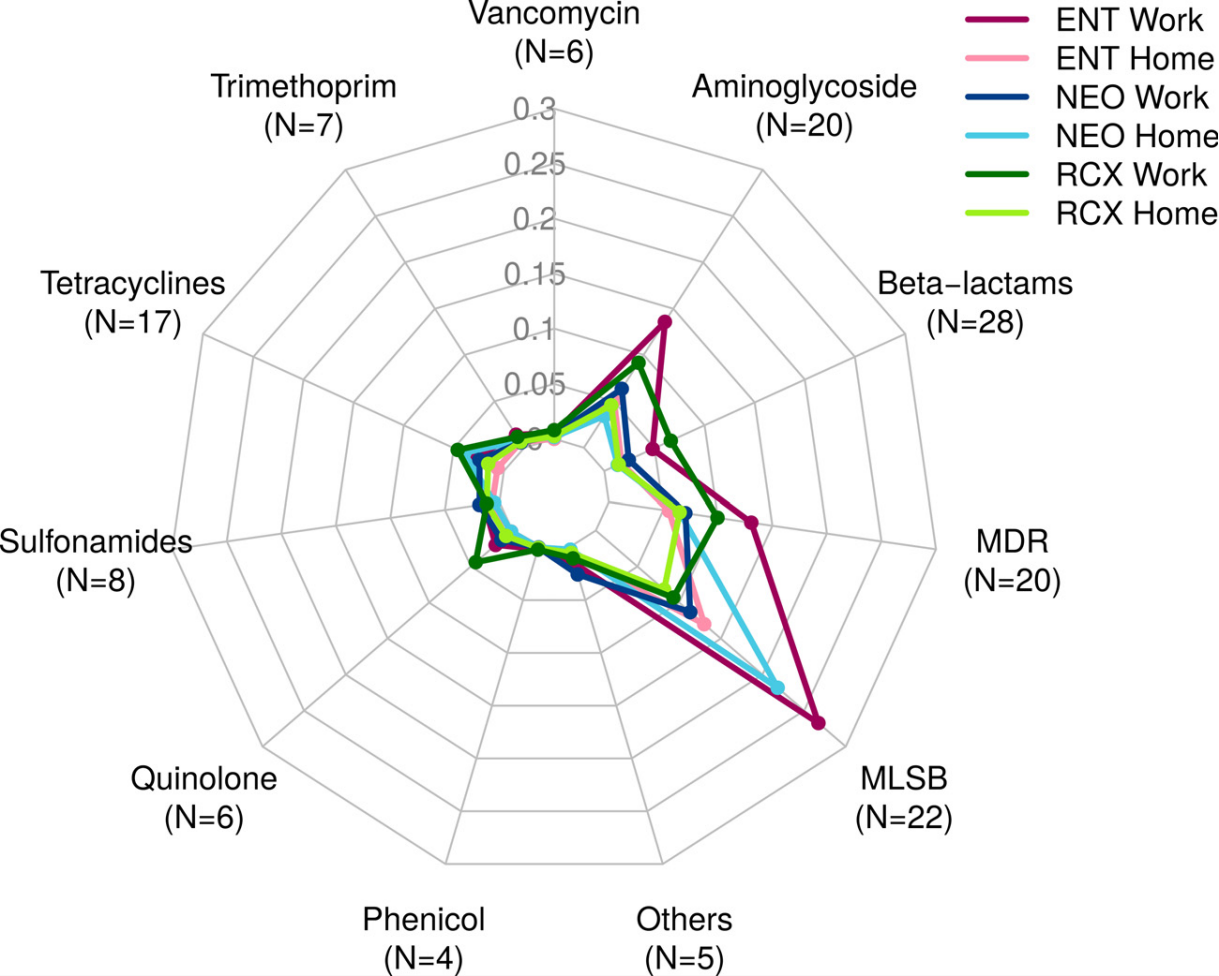
Radar chart containing 143 antibiotic resistance genes (ARGs) detected by HT-qPCR – Resistomap for the dust samples from three workplaces (Work) and households of workers from these workplaces (Home). ARGs were categorized based on the class of antibiotics they confer resistance to. ENT, pediatric hospital; NEO, maternity hospital; and RCX, research center; N, number of ARGs with relative quantity > 0 in any of the six pooled indoor dust samples; MDR, multidrug resistance; MLSB, genes associated with macrolides, lincosamides, and streptogramin B phenotype; HT-qPCR, high-throughput quantitative PCR.

The numbers of ARGs detected by both HT-qPCR – Resistomap analysis (range 47**–**119 ARGs per pooled sample) and WMGS (range 8–23 ARGs per pooled sample) are shown in **Supplementary Table S1**. Out of the 247 ARGs evaluated in total using HT-qPCR, 143 ARGs were detected in the indoor dust samples and could be assigned into eleven categories based on the class of antibiotics they grant resistance to (see **Figure 2**; **Supplementary Table S2**). In comparison, the WMGS analysis revealed only 36 ARGs from nine AR classes (see **Supplementary Table S3**). No ARGs granting resistance to quinolone and sulfonamide antibiotics were detected by WMGS.

Overall, the relative abundance of ARGs detected by HT-qPCR was 1.65 times higher in workplaces compared to households. ARGs associated with the MLSB phenotype were the most prevalent, followed by ARGs for MDR and aminoglycosides. The highest relative abundance of ARGs was detected in the indoor dust samples from the ENT workplace, primarily comprising ARGs of the MLSB phenotype, aminoglycosides, and MDR. Detailed information about the ARG profiles in studied locations, categorized based on the class of antibiotics they confer resistance to, is presented in **Figure 2**.

The highest relative quantity across all pooled samples was recorded for the *ermC_2* gene, which encodes resistance to the macrolide erythromycin (MLSB). It was followed by *ermX_1* (MLSB), *qac* (MDR), *qacA/qacB* (MDR), and *msrA_1* (MLSB).

**Figure 3:**
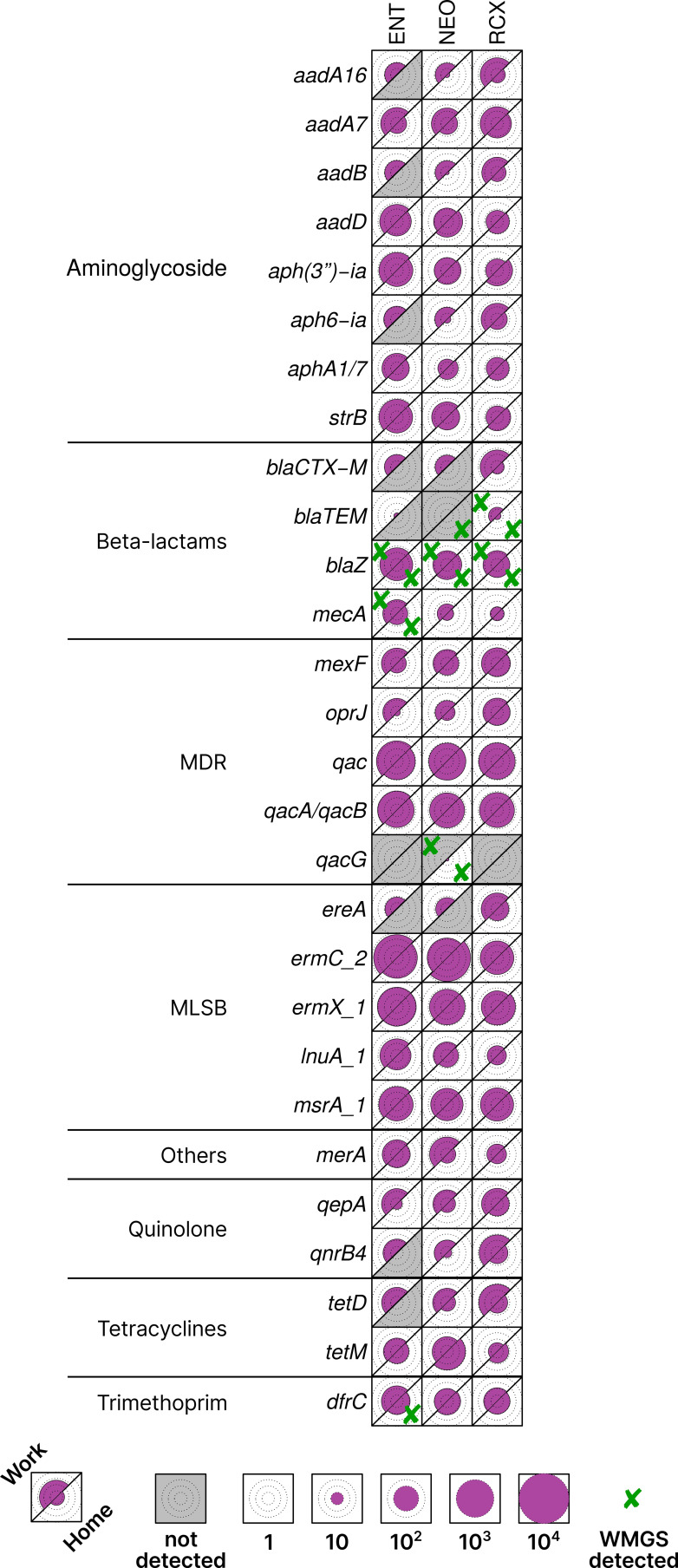
Heatmap of antibiotic resistance genes (ARGs) with > 1% relative quantification in all pooled samples or found in both HT-qPCR and WMGS. Data scaled by the minimal non-zero value across all analyzed samples. The upper left part of each cell corresponds to the workplace (Work) samples, and the lower right part to the household (Home) samples, facilitating easy paired comparison. Circles inside cells indicate orders of magnitude for better readability. Cross marks (✘) denote ARGs that were found also by WMGS. ENT, pediatric hospital; NEO, maternity hospital; and RCX, research center; MDR, multidrug resistance; MLSB, genes associated with the phenotype resistant to macrolides, lincosamides, and streptogramin B; HT-qPCR, high-throughput quantitative PCR; WMGS, whole metagenome shotgun sequencing.

Five ARGs were detected by both HT-qPCR and WMGS: *blaTEM*, *blaZ*, *dfrC*, *mecA*, and *qacG*. 25 ARGs with highest relative quantification across all pooled samples, as well as those found in both HT-qPCR and WMGS, are shown in **Figure 3**. Comprehensive results of HT-qPCR and WMGS are provided in **Supplementary Figure S3**, where, among others, higher relative quantity of *blaNDM* (beta-lactams) can be observed in indoor dust samples from workplaces than in households.

## 4 Discussion

The bacterial diversity and composition of individual indoor dust samples included in this study have been previously analyzed using 16S rRNA amplicon sequencing (Konecna et al., 2023). In this study, we performed two additional types of analysis, a WMGS analysis and an HT-qPCR – Resistomap analysis.

As WMGS was performed in pooled samples due to the HT-qPCR – Resistomap service requirements, it is difficult to compare the bacteriomes found in the pooled samples with the previously published bacteriomes of individual samples detected using 16S rRNA amplicon sequencing. Nevertheless, the bacterial composition identified in the dust samples using both techniques was similar, with high relative abundances of skin-associated bacterial genera, in particular *Corynebacterium*, *Staphylococcus*, and *Cutibacterium/Propionibacterium* (the latter has been recently subsumed under the *Cutibacterium* genus) (Corvec et al., 2019). Higher relative abundances of the genera *Pseudomonas* and *Acinetobacter* have been detected in the maternity hospital. Both *Acinetobacter* and *Pseudomonas* are known to be opportunistic pathogens, frequently associated with AR, especially in healthcare settings. *Acinetobacter* species, particularly *Acinetobacter baumannii*, are recognized for their multidrug resistance, often harboring various ARGs, including those for carbapenems, aminoglycosides, and beta-lactams (Sunenshine et al., 2007; Ayobami et al., 2019; Cain and Hamidian, 2023). *Pseudomonas* species, especially *Pseudomonas aeruginosa*, are also well-documented for their resistance mechanisms, including the production of beta-lactamases, efflux pumps, and the ability to form biofilms (Abdelrahman et al., 2020; Fernández-Billón et al., 2023; Silva et al., 2023; Saeli et al., 2024). These traits contribute to their resistance to multiple classes of antimicrobials, such as beta-lactams, fluoroquinolones, and aminoglycosides.

Eukaryotic DNA, especially human DNA, was significantly prevalent in all dust samples. DNA from pets (dogs) and trees (*Pinus, Picea*) was also relatively abundant in household dust, supporting the hypothesis that households are in greater contact with the surrounding environment due to window ventilation, plants around the house/apartment, and pets. Workplaces are more likely to use HVAC (Heating, Cooling, and Air Conditioning) systems than window ventilation, and the contact of workplaces on higher floors with the external environment is much lower than that of (e.g.) family houses (Barberán et al., 2015; Weikl et al., 2016). The Archea and Viruses domains were significantly less represented than the Eukaryota and Bacteria domains in our study, which is in line with WMGS findings in other studies (Boissy et al., 2014; Sun et al., 2020b; Fu et al., 2021). Among the fungi, the genera *Alternaria*, *Cladosporium*, and *Aspergillus* were the most abundant in the dust samples, which is consistent with findings from other studies investigating the indoor dust mycobiome (Izawa et al., 2020; Khalaf et al., 2022; Chauhan et al., 2023; Nastasi et al., 2024).

In this pilot study, 143 different ARGs were detected by HT-qPCR – Resistomap in indoor dust from three workplaces and 22 households. Similar quantities of ARGs were detected in a study by Zhou et al. (2021), which analyzed the dust resistome in hospital environments (Zhou et al., 2021). The relative quantity of ARGs was higher in the workplaces compared to households. This difference may be caused by a higher level of human occupancy and more frequent use of antimicrobials in the workplaces than in households. ARGs to MLSB were the most prevalent, followed by ARGs to MDR, and to aminoglycosides. The *ermC_2* gene encoding the erythromycin resistance transferase (MLSB group) was the most abundant ARG in the study. It can be found in many Gram-positive bacteria such as *Staphylococcus*, which was highly abundant in all indoor dust samples, as observed in WMGS data as well as in the 16S rRNA amplicon sequencing data published previously by our team (Konecna et al., 2023).

The highest relative abundances of ARGs were detected in the pediatric hospital, suggesting that the hospital environment is a major source of ARGs due to the frequent use of antimicrobials and disinfectants causing selective pressure as reported in many studies (Bonadonna et al., 2017; Christoff et al., 2019; La Fauci et al., 2019). Interestingly, the abundance of ARGs in the samples from the maternity hospital was lower than in the samples from the pediatric hospital. This can be explained by the fact that in the maternity hospital, there is a lower density of sick people needing antimicrobials and an overall lower number of occupants in the building than in the ENT department of the pediatric hospital. Other significant factors may have influenced ARGs abundance, such as the building structure, the materials used in the building, the absence of air conditioning in the maternity hospital, and the distinct disinfecting procedures compared to the pediatric hospital. In the indoor dust samples, especially those collected from workplaces, we detected beta-lactam resistance gene *blaNDM* to the drugs of last resort – carbapenems; these ARGs pose a great risk to healthcare (Codjoe and Donkor, 2017; Brink, 2019; Hammoudi Halat and Ayoub Moubareck, 2020). A recent study by Habibi et al. reported a high prevalence of the ARG *blaIMP*, which is associated with carbapenems, in hospital aerosols. This finding suggests that hospital environments may serve as significant reservoirs for carbapenem-resistant ARGs, with aerosolized dust potentially acting as a vehicle for their dissemination (Habibi et al., 2023).

In households, ARGs associated with MLSB phenotype were the most abundant. A similar trend has been reported by Ding et al (Ding et al., 2020). In addition, they, however, detected a high abundance of ARGs to vancomycin in household dust, which was not replicated in our study. Moreover, the abundances of ARGs in the dust from households of hospital employees were higher than in the samples of dust from households of workers from research center. This can be explained by the transmission of resistant bacteria from hospitals to homes by hospital workers.

The WMGS technique was not able to capture as many ARGs as the HT-qPCR technique and only five ARGs were detected in the same pooled sample by both techniques. The advantage of the WMGS technique is that, in addition to the detection and quantification of ARGs, it also allows the analysis of the metagenome and metabolic pathways by mapping the sequence reads to sequences in reference databases. On the other hand, HT-qPCR certainly provides better detection limits than the WMGS approach due to the targeted analysis of selected genes. The advantage of SmartChip HT-qPCR technique lies in the simultaneous qualitative as well as quantitative analysis of a high number of ARGs (up to 384) within a short time; however, the limitation lies in the fact that only previously described ARGs can be detected, and information on the host microorganism carrying ARGs cannot be obtained (van Schaik, 2015; Kim and Cha, 2021; Daw Elbait et al., 2024).

Our pilot study aimed to describe the abundance of ARGs in indoor environments where people spend most of their time, such as workplaces and households, providing preliminary insights into the resistome structure in these settings. Unlike the study by Wang et al (Wang et al., 2020), which evaluated the resistome in human and animal gut microbiomes using metatranscriptomic profiling to capture active ARG expression, we did not focus on gene expression which is a critical factor in assessing the risk and impact of ARGs. Nevertheless, our findings offer valuable information on the genetic potential of the resistome in households and workplaces—indoor environments that have not been as extensively studied as human and animal gut resistomes.

The main limitation of this pilot study lies in the low amount of DNA in individual analyzed samples, which led to the necessity of their pooling to support HT-qPCR analysis (and, in effect, to the low number of analyzed samples). Collecting a higher amount of dust from each location would support the extraction of a higher amount of bacterial DNA from individual samples and, in turn, enable analysis of a higher number of samples pairing individual workers and their household/home environments instead of a general pooled sample; this is something we recommend for future studies. On the other hand, based on the available literature, this is the first comparative study describing the indoor dust resistomes from workplaces and homes of the employees from these workplaces.

## Data Availability

The datasets presented in this study can be found in online repositories. The names of the repository/repositories and accession number(s) can be found in the article/**Supplementary Material**.
