# Supplementary figures and images for: Resistome in the indoor dust samples from workplaces and households: a pilot study
Source: Front Cell Infect Microbiol. 2024 Dec 3;14:1484100. doi: 10.3389/fcimb.2024.1484100 (PMC11649746; doi:10.3389/fcimb.2024.1484100)

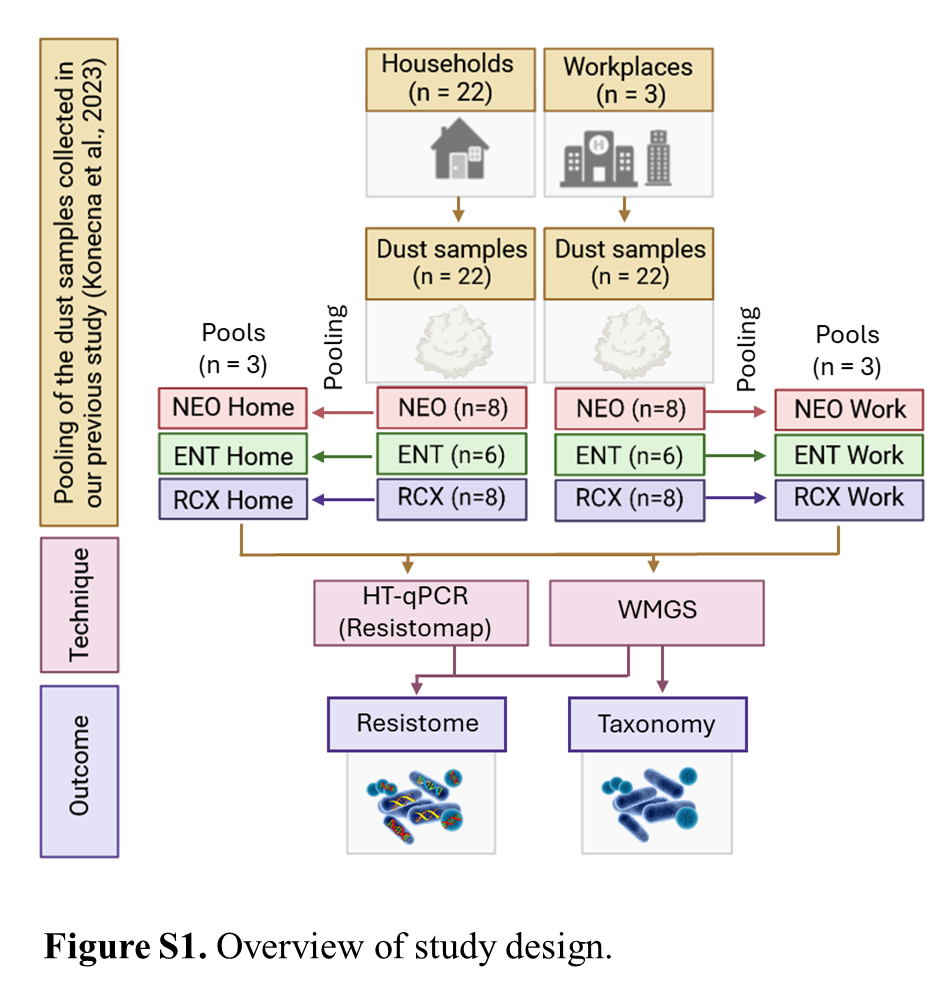

Supplement: Supplementary file 1 [file Image1.png]
