# Supplementary material for: Resistome in the indoor dust samples from workplaces and households: a pilot study
Source: Front Cell Infect Microbiol. 2024 Dec 3;14:1484100. doi: 10.3389/fcimb.2024.1484100 (PMC11649746; doi:10.3389/fcimb.2024.1484100)
Supplement: Supplementary file 2 [file Image2.pdf]

nf-core/mag params json:


```
{
   "input": "samplesheet.csv",
   "outdir": ".\/results",
   "save_clipped_reads": true,
   "reads_minlength": 50,
   "fastp_cut_mean_quality": 25,
   "skip_spadeshybrid": true,
   "skip_spades": true,
   "skip_prodigal": true,
   "skip_binning": true,
   "skip_binqc": true
}
```


nf-core/funcscan params json:


```
{
   "input": "samplesheet.csv",
   "outdir": ".\/results",
   "run_arg_screening": true,
   "arg_skip_deeparg": true,
   "arg_skip_rgi": true,
   "arg_skip_fargene": true,
   "arg_hamronization_summarizeformat": "tsv"
}
```


Supplementary Figure S2. Bioinformatics analysis parameters.
