# Supplementary material for: Resistome in the indoor dust samples from workplaces and households: a pilot study
Source: Front Cell Infect Microbiol. 2024 Dec 3;14:1484100. doi: 10.3389/fcimb.2024.1484100 (PMC11649746; doi:10.3389/fcimb.2024.1484100)
Supplement: Supplementary file 3 [file Image3.pdf]

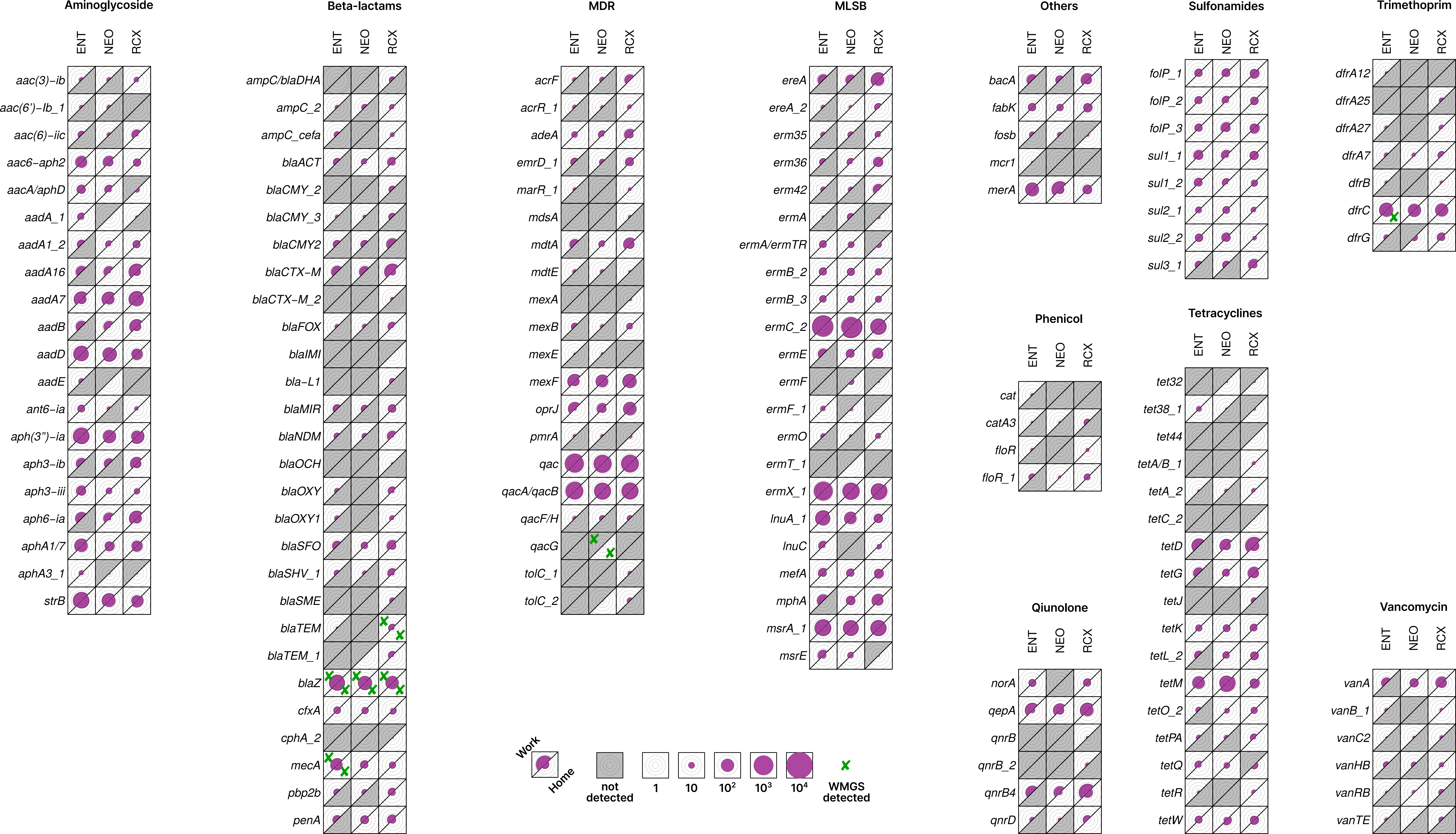

Aminoglycoside
ENT
NEO
RCX
aac(3)-ib
aac(6')-Ib_1
aac(6)-iic
aac6-aph2
aacA/aphD
aadA_1
aadA1_2
aadA16
aadA7
aadB
aadD
aadE
ant6-ia
aph(3")-ia
aph3-ib
aph3-iii
aph6-ia
aphA1/7
aphA3_1
strB
Beta-lactams
ampC/blaDHA
ampC_2
ampC_cefa
blaACT
blaCMY_2
blaCMY_3
blaCMY2
blaCTX-M
blaCTX-M_2
blaFOX
blaIMI
bla-L1
blaMIR
blaNDM
blaOCH
blaOXY
blaOXY1
blaSFO
blaSHV_1
blaSME
blaTEM
blaTEM_1
blaZ
cfxA
cphA_2
mecA
pbp2b
penA
MDR
acrF
acrR_1
adeA
emrD_1
marR_1
mdsA
mdtA
mdtE
mexA
mexB
mexE
mexF
oprJ
pmrA
qac
qacA/qacB
qacF/H
qacG
tolC_1
tolC_2
MLSB
ereA
ereA_2
erm35
erm36
erm42
ermA
ermA/ermTR
ermB_2
ermB_3
ermC_2
ermE
ermF
ermF_1
ermO
ermT_1
ermX_1
lnuA_1
lnuC
mefA
mphA
msrA_1
msrE
Others
bacA
fabK
fosb
mcr1
merA
Phenicol
cat
catA3
floR
floR_1
Qiunolone
norA
qepA
qnrB
qnrB_2
qnrB4
qnrD
Sulfonamides
folP_1
folP_2
folP_3
sul1_1
sul1_2
sul2_1
sul2_2
sul3_1
Tetracyclines
tet32
tet38_1
tet44
tetA/B_1
tetA_2
tetC_2
tetD
tetG
tetJ
tetK
tetL_2
tetM
tetO_2
tetPA
tetQ
tetR
tetW
Trimethoprim
dfrA12
dfrA25
dfrA27
dfrA7
dfrB
dfrC
dfrG
Vancomycin
vanA
vanB_1
vanC2
vanHB
vanRB
vanTE
Work
Home
not detected
1
10
10²
10³
10⁴
WMGS detected

Supplementary Figure S3. Heatmap of antibiotic resistance genes (ARGs) in all pooled samples found by HT-qPCR (relative quantity) and/or WMGS (presence). Data scaled by the minimal non-zero value. The upper part of each cell corresponds to household samples (Home) and the lower diagonal part corresponds to workplace samples (Work), facilitating easy paired comparison. Circles inside cells indicate orders of magnitude for readability. Cross marks (✗) denote ARGs found in WMGS.

ENT, pediatric hospital; NEO, maternity hospital; and RCX, research center; MDR, multidrug resistance; MLSB, genes associated with macrolides, lincosamides, and streptogramin B phenotype; HT-qPCR, high-throughput quantitative PCR; WMGS, whole metagenome shotgun sequencing
